# Combining Genome-Wide Information with a Functional Structural Plant Model to Simulate 1-Year-Old Apple Tree Architecture

**DOI:** 10.3389/fpls.2016.02065

**Published:** 2017-01-12

**Authors:** Vincent Migault, Benoît Pallas, Evelyne Costes

**Affiliations:** INRA, UMR 1334 AGAP, Equipe Architecture et Fonctionnement des Espèces FruitièresMontpellier, France

**Keywords:** FSPM, genome wide prediction, RR-BLUP, temperature effect, branching

## Abstract

In crops, optimizing target traits in breeding programs can be fostered by selecting appropriate combinations of architectural traits which determine light interception and carbon acquisition. In apple tree, architectural traits were observed to be under genetic control. However, architectural traits also result from many organogenetic and morphological processes interacting with the environment. The present study aimed at combining a FSPM built for apple tree, MAppleT, with genetic determinisms of architectural traits, previously described in a bi-parental population. We focused on parameters related to organogenesis (phyllochron and immediate branching) and morphogenesis processes (internode length and leaf area) during the first year of tree growth. Two independent datasets collected in 2004 and 2007 on 116 genotypes, issued from a ‘Starkrimson’ × ‘Granny Smith’ cross, were used. The phyllochron was estimated as a function of thermal time and sylleptic branching was modeled subsequently depending on phyllochron. From a genetic map built with SNPs, marker effects were estimated on four MAppleT parameters with rrBLUP, using 2007 data. These effects were then considered in MAppleT to simulate tree development in the two climatic conditions. The genome wide prediction model gave consistent estimations of parameter values with correlation coefficients between observed values and estimated values from SNP markers ranging from 0.79 to 0.96. However, the accuracy of the prediction model following cross validation schemas was lower. Three integrative traits (the number of leaves, trunk length, and number of sylleptic laterals) were considered for validating MAppleT simulations. In 2007 climatic conditions, simulated values were close to observations, highlighting the correct simulation of genetic variability. However, in 2004 conditions which were not used for model calibration, the simulations differed from observations. This study demonstrates the possibility of integrating genome-based information in a FSPM for a perennial fruit tree. It also showed that further improvements are required for improving the prediction ability. Especially temperature effect should be extended and other factors taken into account for modeling GxE interactions. Improvements could also be expected by considering larger populations and by testing other genome wide prediction models. Despite these limitations, this study opens new possibilities for supporting plant breeding by *in silico* evaluations of the impact of genotypic polymorphisms on plant integrative phenotypes.

## Introduction

Tree architecture plays a key role in collecting light and assimilating carbon and thus affects plant growth and yield (Valladares et al., 2002; Niinemets, 2010). Moreover, by modifying plant microclimate, it also determines many characteristics of fruit quality and can affect the development of orchard pests and diseases (Lauri and Costes, 2009). Previous studies have demonstrated that, across vascular plants, architecture exhibits remarkable regularities (Hallé et al., 1978; Sussex and Kerk, 2001) which are assumed to result from genetic control (Reinhardt and Kuhlemeier, 2002). Even though genetics and breeding aim at selecting optimal combinations of traits (a genotype with such optimal characteristics being considered as an ideotype; Donald, 1968), the optimization of plant architecture is not straightforward because it results from many morphological and physiological processes interacting with the environment. In addition, defining an ideotype for a given species and for particular environmental or agronomic conditions can be extremely time- and resource consuming especially in perennials which characteristics may change over years. Modeling appears as an appropriate tool for the prediction and simulation of tree architecture and related properties in order to explore possible ideotypes.

Functional-structural plant models (FSPM) have been developed for analyzing the relationships between plant architecture, light interception, microclimate, and physiological processes such as carbon assimilation or water losses by transpiration (Vos et al., 2010; DeJong et al., 2011). These models combine a 3D representation of plant architecture with mathematical formalisms describing physiological functions (photosynthesis, carbon allocation…) at different scales of plant organization (from organs to plant). This kind of models has been developed for different annual (e.g., Fournier et al., 2003; Evers et al., 2007), or perennial crops (e.g., Perttunen et al., 1998; Allen et al., 2005; Costes et al., 2008). Most of them have been calibrated and validated on one or a few genotypes with contrasting architectures. However, there is strong evidence that components and processes responsible for plant architecture are highly heritable and under genetic control (Wu and Stettler, 1994; Wu, 1998; De Wit et al., 2002; Perez et al., 2016). This opened new perspectives for integrating architectural traits in breeding programs (Wu, 1998; Rötter et al., 2015).

Previous studies have included the genetic variability in FSPMs by estimating physiological or morphological parameters using QTL information (Letort et al., 2008; Xu et al., 2012). More precisely, QTL effects have been used to predict the values of some FSPM input parameters such as those determining organ sing strength. QTL effects have been estimated on virtual recombinant populations (Letort et al., 2008). However, these methods could be applied if QTLs are detected for model parameters whereas architectural traits revealed high polygenic determinisms (Wu and Stettler, 1994; Segura et al., 2008). Concomitantly, the development of high density genetic maps with single nucleotide polymorphism (SNP) markers have allowed the emergence of genome wide prediction models which aims at predicting genotypic values based on whole genome information (Heffner et al., 2009; Kumar et al., 2012). The statistical challenge of the genome wide prediction models is to estimate in a regression model the effects of a number of markers larger than the number of individuals, this leading to over-fitting problems. Statistical methods have been developed to reduce the risk of over-fitting (de los Campos et al., 2013) by applying a selection of variables and/or a shrinkage parameter on marker effects. Genome wide prediction models used a mixed model approach and marker effects are estimated according to a prior distribution. This prior distribution can be a Gaussian distribution in the Ridge Regression BLUP method (Endelman, 2011), a double exponential or t distributions in Bayesian approaches (de los Campos et al., 2013).

The aim of the present study was to combine a FSPM previously built for apple tree (MAppleT, Costes et al., 2008) with genetic determinisms of architectural traits in a bi-parental segregating population. This population has been previously studied and showed a strong heritability of architectural traits (Segura et al., 2006, 2008). Our objective was to explore the feasibility of combining the effects of SNP markers on model parameters related to architectural traits with MAppleT in order to simulate tree architecture.

MAppleT simulates apple tree above-ground development over years (Costes et al., 2008), by combining Markovian models to simulate the dynamic of tree topology and a bio-mechanical model to simulate the geometry (branch angle and bending; Jirasek et al., 2000; Alméras et al., 2002). Presently, MAppleT does not simulate any environmental effect on growth processes and does not include the simulation of carbon source-sink relationships or water fluxes within the tree. Primary growth is modeled based on the phyllochron (time between the appearance of two consecutive leaves) which is considered as a constant value and the final size of organs (internode, leaf) is an input parameter of the model. Previous studies showed that changing the values of these architectural traits in MAppleT, considering the range of values observed in the segregating population mentioned above, modifies the light interception of the simulated trees (Da Silva et al., 2014).

We focused on the vegetative development during the first year of apple tree growth which is a critical period for architecture establishment. During this initial growth period, apple tree develops a main stem with immediate laterals (or sylleptic; Segura et al., 2006). Therefore, the main variables of interest for simulating primary growth and branching at this stage are the phyllochron, the internode length, the individual leaf area, and the probability of appearance of the sylleptic laterals along the main stem. Some relations between these variables have been observed. Notably, the phyllochron is assumed to modulate sylleptic branching since the number of sylleptic laterals is often higher when the phyllochron is low during the season (Remphrey and Powell, 1985; Powell, 1991; Génard et al., 1994). Moreover, temperature affects the phyllochron of some fruit species such as kiwifruit or grapevine (Morgan et al., 1985; Pallas et al., 2008). For peach tree, the phyllochron could be modeled as a function of thermal time (TT; Kervella et al., 1995; Lescourret et al., 1998) even if temperature was not the only factor influencing the phyllochron when trees are grown in open field conditions (Davidson et al., 2015).

In this study, we simulated 1-year-old apple tree architectures depending on the genotype and air temperature over a growing season. This approach was built through four sub-objectives: (i) modeling the dynamic of phytomer emergence and the subsequent sylleptic branching depending on TT, (ii) estimating key MAppleT parameters based on SNP marker effects, (iii) integrating the environmental and the marker effects in MAppleT to simulate genotype development based on genetic and climatic information, (iv) validate the method by comparing simulation outputs with observed data.

## Materials and Methods

### Plant Material, Phenotyping, and Genotyping

The study was carried out on 116 genotypes corresponding to an apple tree (Malus × Domestica) F1 progeny issued from a “Starkrimson” × “Granny Smith” cross. The parents were selected for their contrasted architecture and bearing behavior. Indeed, the ‘Starkrimson’ maternal parent displays an erect growth habit with many short shoots and a tendency to irregular bearing (Lespinasse, 1992). Conversely, the ‘Granny Smith’ pollen parent displays a weeping habit with long shoots and fruit-bearing regularity (Segura et al., 2007, 2008).

Two field experiments were planted in 2007 (Experiment 1) and 2004 (Experiment 2) at the Melgueil INRA Montpellier experimental station (France, 43°36′N 3°58′E). In both experiments, two replicates of each genotype were grafted on Pajam 1 rootstock and planted 5 m × 1.5 m apart in a north-south orientation. Throughout the experiments, the 232 trees were well-watered and not pruned.

In each experiment, the topology of each tree was described as explained in Segura et al. (2006) at the end of the first growth season and recorded in Multiscale Tree Graph format (MTG, Godin and Caraglio, 1998). The trunk length, the mean internode length on the trunk, the number of sylleptic laterals, and their position along the trunk were extracted from the MTG. In Experiment 1, the number of newly emerged leaves on trunk was also counted weekly, from July to the end of growth in September (11 dates of measurement). When trees were 3-year-old in Experiment 1, the maximal individual leaf areas along four randomly chosen shoots were measured on each tree at the end of the growing season (end of September 2009). This maximal leaf area has been already observed to be highly heritable and to display lower variability than individual leaf areas along the trunk (Lopez et al., 2015).

The population was genotyped with the Infinium^®^ 20K SNP array (Bianco et al., 2014) at the Fondazione Edmund Mach according to the procedures described by Antanaviciute et al. (2012) and Chagné et al. (2012). Among the 6849 polymorphic SNPs, 3123 were used in this study, after a careful checking of their robustness consistency (Van de Weg et al., 2013; Di Guardo et al., 2015) and recombination pattern (Allard et al., 2016). In particular, all markers which were not strictly bi-allelic in at least one of the parent were discarded. We removed these markers because there were not informative to evaluate genetic effects in quantitative genetic studies. Indeed, in that case, all the individuals of the progeny had the same alleles at these markers.

### Modeling Temperature Impact on Leaf Emergence Rate and Sylleptic Branching

Nine linear mixed models (see **Supplementary Material 1.1**) were compared to analyze the effect of temperature on the daily rate of leaf emergence (RLE) and its genetic variability depending on temperature. Since we did not want to increase the number of parameters in the MAppleT, the BIC was chosen for model selection because it is known to be more adequate for selecting models with a few number of parameters compared to the AIC (Davidson and Mackinnon, 2004). The two first models did not include temperature effect and assumed a constant RLE throughout the growing season. Two different methods were used to calculate TT based on either the daily mean temperature (T*_Mean_*) or a single sine method (T*_SSM_*) using 7 and 35°C as lower and upper threshold as in Lescourret et al. (1998) on peach tree. In the subsequent analyzes, the RLE was expressed in growing degree-day (RLE*_GDD_*) and computed as the slope of the relationship between TT using T*_SSM_* and the number of emitted leaves.

To account for the relationship between the parent shoot growth and the appearance of sylleptic lateral at a given node, each day the probability of producing a sylleptic lateral, noted *P_d_*, was assumed to be linearly dependent on the *RLE*. In this study and according to Peyhardi et al. (2013), we considered the values of *RLE* during the *n_days_* days before leaf emergence of the considered metamer on which sylleptic lateral development can occur. Moreover, we assumed that a sylleptic lateral only appears at a given rank (called *r_k_*) below the terminal apex, in order to represent the inhibition of axillary bud outgrowth by the apical meristem (Cline, 2000; Beveridge, 2006). In the model, the probability of sylleptic lateral emergence was computed when a new leaf was emitted by the terminal apex. The probability of appearance of a sylleptic lateral at a node of rank *k - r_k_* and at day *d* was thus expressed as follows:

$$P_{d}(Sylleptic\,branching(k-r_{k})|Phytomer\,creation\left(k\right))=a_{syll}\times \frac{1}{n_{days}}\overset{d}{\underset{i={d-n}_{days}}{\Sigma }}{RLE}_{i}$$

where *a_syll_* is the coefficient of the linear relation between *P_d_* and the mean value of *RLE* during the *n_days_* previous days.

The values of *r_k_* and *n_days_* were determined with a virtual experiment in which tree development was simulated with MAppleT using different values, 5, 10, 15, and 20 for *r_k_* and 3, 7, and 10 for *n_days_*, respectively (see **Supplementary Figure 1**). The best combination of parameters (20 *LD* and 7 *n_days_*) was defined as the combination which minimizes the root mean squared error (RMSE) between observed and simulated distributions of sylleptic laterals along the trunk. *r_k_* and *n_days_* were estimated on the mean distribution of sylleptic laterals considering all genotypes together. Indeed, since sylleptic branching is a rare event, the number of replicates (only two) was not enough to get a consistent representation of the distribution of sylleptic laterals along the trunk for each genotype of the population. Values of *r_k_* and *n_days_* were thus considered as identical for each genotype.

**FIGURE 1:**
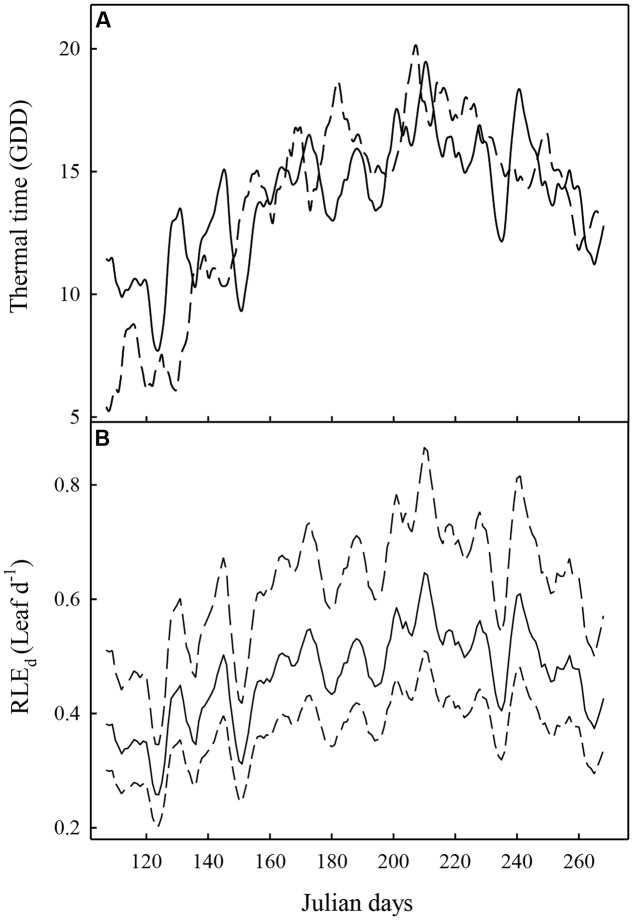
**Evolution of the thermal time** **(A)** and the rate of leaf emergence per day (*RLE_d_*) **(B)** averaged over 5 days (from 2 days before to 2 days after each day in abscissa), the two experiments (Experiment 1: full line; Experiment 2: dashed line). In **(B)**, the mean value of *RLE*_d_ on the 116 hybrids is represented with a full line. The dashed lines represent the maximum and minimum *RLE_d_* values recorded on the population.

### Estimation of MAppleT Parameter Values

We focused on four parameters used in MAppleT, (i) *RLE_GDD_*, (ii) the length of individual internode (*IN_length*), (iii) the area of individual leaf (*Leaf_area*), and (iv) *a_syll_*. In MAppleT, these parameters are the main parameters driving primary growth and branching. The other parameters that can affect primary tree growth in MAppleT are the parameters related to the dynamics of organ expansion but we did not consider them in this study because they had no impact on the final architecture (all the organs end their growth at the end of the growth season) and because they are difficult to estimate experimentally on a large range on genotypes.

Values of these four parameters were estimated on each tree in Experiment 1. *IN_length* was the mean internode length recorded at the end of the experiment. *Leaf_area* was estimated as the mean leaf area of the four leaves measured at the end of Experiment 1. These dimensions were then considered in MAppleT as the final dimensions of internodes and leaves for all the metamers along the trunk and branches expect for the preformed metamers (eight first leaves). In MAppleT, organ expansion is modeled as a logistic growth functions and the value of this function reaches *Leaf_area* or *IN_Length* at the end of the organ growth period (Da Silva et al., 2014).

The value of *a_syll_* was estimated for each tree as a function of the total number of sylleptic laterals (*N_syll_*), the RLE*_GDD_* and the sum of the daily thermal time (*TT_d_*) during the growing season (15 April–30 September; see more information in **Supplementary Material 1.2**), as follows:

$$a_{syll}=\frac{N_{syll}}{{\left({RLE}_{GDD}\right)}^{2}\times \Sigma_{d}^{d_{e}}=d_{b}(\frac{1}{n_{days}}\Sigma_{i}^{d}={d-n}_{days}{TT}_{i})\times {TT}_{d}}$$

where *d_b_* is the day when the node of rank *r_k_* appears on tree, *d_e_* is the day of the end of growing season.

The value of *RLE*_GDD_** for each individual was estimated from the linear model between RLE and TT that minimized the BIC (see **Supplementary Table 1**).

### Analyses of the Genetic Variability, Correlations, and Heritability Values of MAppleT Parameters and Architectural Variables

Analyses of genetic variability and heritability were performed on the four MAppleT parameters and on three architectural traits directly measured on trees: the length of trunk (*Trunk_length*), the total number of leaves (*Nb_Leaves*), and the number of sylleptic laterals (*Nb_syll*). Moreover, as *a_syll_* distribution was not Gaussian, its logarithm, called *loga_syll_*, which was normally distributed, was used for the genetic analyses.

The significance of the genotypic effect on the four parameters and on the three integrative variables was estimated, for both experiments separately, using a linear mixed model with genotype as random effect as follows:

$$P_{ij}=\mu +G_{i}+e_{ij}$$

where *P_ij_* is the phenotype value of the *j^th^* replicate of genotype *i*, μ is the overall mean of the progeny, *G_i_* is the random effect of the genotype *i*, and *e_ij_* is the gaussian residual. This model was fitted on data using the *lmer* procedure of the *lme4* package of R software. The mean broad-sense heritability was calculated as:

$$h_{b}^{2}=\frac{\sigma_{G}^{2}}{\sigma_{G}^{2}+\frac{\sigma_{e}^{2}}{n}}$$

where $\sigma_{G}^{2}$ is the genotypic variance, $\sigma_{e}^{2}$ is the residual variance, and *n* is the number of replicates per genotype. In order to analyze the genetic variability on the two experiments together, the following linear mixed model using years as fixed effect and genotype as random effect was used.

$$P_{ijk}=\mu +A_{i}+G_{j}+I_{ij}+e_{ijk}$$

where *P_ijk_* is the phenotype value of the *k^th^* replicate of genotype j in the year *i*, μ is the overall mean of the progeny over the 2 years, *A_i_* is the fixed effect of the year *i*, *G_j_* is the random effect of the genotype *j*, *I_ij_* is the random effect of the interaction between the effects of the year *i* and the genotype *j* and *e_ijk_* is the gaussian residual. The mean broad-sense heritability over the 2 years was calculated for the whole data as:

$$h_{b}^{2}=\frac{\sigma_{G}^{2}}{\sigma_{G}^{2}+(\frac{\sigma_{i}^{2}}{a})+\left(\frac{\sigma_{e}^{2}}{n\times a}\right)}$$

where $\sigma_{G}^{2}$ is the genotypic variance, $\sigma_{I}^{2}$ is the interaction variance, $\sigma_{e}^{2}$ is the residual variance, *a* is the number of years, and *n* is the number of replicates. The significance of the year effect was evaluated using *anova* procedure of the *lmerTest* package of R software.

For each heritability value, associated confidence intervals were calculated as defined in Knapp et al. (1985). The phenotypic and genotypic correlations between MAppleT parameters and architectural traits were calculated for each experiment separately. Correlations between the two experimental dataset were calculated for the three architectural traits, only. The significance of the correlations was tested with a Pearson’s test.

### Genomic Prediction of MAppleT Parameter Values

Following Xu et al. (2011), QTL were detected on BLUPs for the four studied parameters using the interval mapping method within the MapQTL software (Van Ooijen et al., 2002). The logarithm of the odds (LOD) at which QTL was declared significant was determined according to a genome wide error of 0.05 over 2000 permutations of the data. A QTL was considered as significant when the associated LOD was higher than 4.

As polygenic determinisms were expected, a genomic prediction method based on Ridge Regression was used after this QTL analysis (Whittaker et al., 2000; Meuwissen et al., 2001). More precisely, this method estimates infinitesimal loci effects assuming a shrinkage parameter equals to the ratio of the residual to the environmental variance using the following mixed effect model:

$$Y=\mu +X_{g}+e$$

where *Y* are the phenotypic values (232 × 1; two replicates per genotype), μ is the overall mean of phenotypic value, *X* (232 × 2160) is the matrix of markers for each individual, *g* (2160 × 1) is the marker effects which are assumed to be normally distributed $\left(g∼N\left(0,I\sigma_{g}^{2}\right)\right)$, and *e* (232 × 1) is the residual effects $\left(g∼N\left(0,I\sigma_{e}^{2}\right)\right)$. In this study, we only kept 2160 markers among the 3123 SNPs available in the genetic map. These 2160 markers correspond to the markers that were heterozygous for one of the two parents, only. As a consequence, the matrix of markers includes one kind of heterozygous (A/B) and one kind of homozygous markers (A/A), only. In the matrix of markers, a locus was then coded -1 when the two alleles were different (A/B), and was coded 1 when the two alleles were similar (A/A), whatever the heterozygous parent. Marker effect estimations were performed on phenotypic values collected in Experiment 1 data using the mixed.solve procedure of the rrBLUP package of R software (Endelman, 2011).

We estimated the genetic values of each parameter (*G*, (116×1)) for all genotypes in the population using the estimated marker effects (*ĝ*):

G=μ+Wg^

with *W* the matrix of markers for each genotype (116 × 2160). In order to predict the phenotypic values of MAppleT parameters, markers effects were multiplied by the ratio between the standard deviation of observed phenotypes and the standard deviation of the genotypic predictions.

g2=sd(Y)sd(G)×g^

The phenotypic values of MAppleT parameters (*P*, (116 × 1)) was calculed for each parameters and for all the genotypes as follows:

$$P=\mu +W_{g2}$$

The accuracy of the genome wide prediction model was assessed by testing the correlations between estimated and observed values of the MAppleT parameters, in three different ways. First, the accuracy of the prediction model was determined using all genotypes to estimate parameter values. Second, and following previous studies (Kumar et al., 2012; Fodor et al., 2014), the accuracy of the prediction was evaluated using a 10-fold cross validation. For this, genotypes were randomly assigned to one of the 10 equal folds. Each fold (validation population, 10% of the population) was predicted using marker effect estimations using the nine other folds (training population). In this case, the accuracy was calculated as the mean value of the correlations between predicted and observed phenotypes obtained for the 10 folds. Lastly, we also tested the accuracy of the prediction with a “leave-one-out” cross validation. In this method, each phenotype was predicted using marker effects estimated on the whole population without this genotype. The accuracy was calculated as the correlation between predicted and observed phenotypes on the whole population.

### Implementation of Markers Effects in MAppleT and Output Evaluation

The values of the four parameters (*IN_length*, *Leaf_area*, *RLE*_GDD_**, and *a_syll_*) for all the genotypes were calculated using the genome wide prediction model based on the matrices of marker effects and genotypes. Simulations were then performed with MAppleT with the environmental conditions of both experiments.

For validating the approach, the values of the three integrated architectural traits (*Nb_Leaves, Trunk_length*, and *Nb_syll*) were compared with observed values. Since the sylleptic branching model is stochastic, different values were obtained for the different simulations with the same set of genotypic parameters. The observed number of sylleptic laterals per genotype was thus compared with the mean number of sylleptic obtained after running five simulations.

The validation was performed in two consecutive steps. First, simulation outputs were compared with data from Experiment 1, which was used to parameterize the model, in order to evaluate the model capacity to simulate the genotypic variability of whole tree development based on markers effects. Second, the comparison was performed on data from Experiment 2 which were not used to parameterize the model in order to evaluate its performance under different environmental conditions. Simulations were run from budburst until the end of the growing season. In both experiments and accordingly to what is observed in the experimental site, budburst date, and the end of the growing season were set to April, 15 and September, 30, respectively.

The correlation coefficient, the *RMSE*, the relative RMSE (*nRMSE*), and the bias (*BIAS*) were used to compare simulations (*s_i_*) and observations (*o_i_*):

$$RMSE=\sqrt{\frac{1}{n}\overset{n}{\underset{i=1}{\Sigma }}{\left(O_{i}-S_{i}\right)}^{2}\,}$$

$$nRMSE-RMSE/\overset{¯}{o}$$

$$BIAS=\frac{1}{n}\overset{n}{\underset{i=1}{\Sigma }}\left(S_{i}-O_{i}\right)$$

## Results

### Climatic Data and Phenotypic Traits

The analysis of temperature effect on the RLE showed that the mixed linear model 4 which includes the effect of TT computed with the single sine method, *TT* (see **Supplementary Table 2**) had the lowest BIC. Although BIC values between the different models were close, this sustains the hypothesis of a significant impact of temperature variations on RLE. Moreover, model 4 includes a genetic effect (β_ι_) on the slope between RLE and TT (RLE*_GDD_*) which also suggests that the slope values were genetically dependent.

The cumulate daily thermal time (*TT*) during the growing season (15 April–31 September) was almost similar between the two experiments, even though slightly higher in Experiment 1 than in Experiment 2 (2278 and 2234 GDD, respectively). However, the annual dynamics were different. Indeed, daily thermal time was strongly higher during the first 30 days and was slightly lower during summer (June, August, and September) in Experiment 1 compared to Experiment 2 (**Figure 1**). For the two experiments, the highest GDD was observed at the end of July.

As the leaf emergence rate (RLE*_d_*) and the probability of sylleptic lateral emergence directly depends on TT in our model, the evolution of these two variables followed the daily thermal time. The mean value of the leaf emergence rate expressed in growing degree-day (*RLE*_GDD_**) and estimated on data from Experiment 1 was 0.033 Leaf.GDD^-1^ which corresponds to a duration between the emergence of two leaves of 30 GDD (∼3 days during the first growing month and 2 days during summer). The mean value of the estimated parameter *loga_syll_* was -1.25. The mean value of log*a_syll_* corresponded to a probability of emergence of a sylleptic lateral on each metamer of 0.095 during first growing months and of 0.143 during summer.

Compared to Experiment 1, phenotypic values observed in Experiment 2 were on average 5% higher for the number of leaves (*Nb_Leaves*), 12% lower for the trunk length (*Trunk_length*), and 48% higher for the number of sylleptic laterals (*Nb_syll*) and these differences were significant (**Table 1**). It must be noticed that the higher number of leaves in Experiment 2 compared to Experiment 1 is contradictory with the corresponding value of cumulated daily thermal time which was lower in Experiment 2.

**Table 1:** Mean value, standard deviation, mean heritability value ($h_{b}^{2}$) with confidence interval (CI) indicated in brackets for five measured variables (*Nb_Leaves*, *Trunk_length*, *Nb_Syll*, *IN_length*, and *Leaf_area*) and two estimated model parameters (*RLE*_GDD_**, *loga_syll_*) on trunks of 116 1-year-old hybrid apple trees, in experiment 1.

Variable	Experiment 1		Experiment 2		All data^†^	Year effect
				
	Mean ±*SD*	$h_{b}^{2}$ (CI)	Mean ±*SD*	$h_{b}^{2}$ (CI)	$h_{b}^{2}$ (CI)	
Nb_Leaves	83.54 ± 9.38	0.50 (0.28,0.65)	87.86 ± 13.20	0.51 (0.29,0.66)	0.38 (0.11,0.57)	^∗∗∗^
Trunk_length (cm)	168.4 ± 28.5	0.61 (0.44,0.73)	148.4 ± 34.5	0.56 (0.36,0.69)	0.61 (0.44,0.73)	^∗∗∗^
Nb_Syll	12.38 ± 10.33	0.74 (0.63,0.82)	18.38 ± 12.21	0.70 (0.57,0.79)	0.49 (0.26,0.64)	^∗∗∗^
IN_length (cm)	2.02 ± 0.28	0.86 (0.80,0.90)				
Leaf_area (cm^2^)	25.54 ± 5.99	0.40 (0.14,0.59)				
RLE*_GDD_* (Leaf.GDD^-1^)	0.033 ± 0.003	0.59 (0.40,0.71)				
log*a_syll_*	-1.25 ± 1.03	0.72 (0.59,0.80)				


*^†^For analysis on the whole dataset, heritability were estimated using the mixed model with year as fixed effect.*

*Stars indicated the significance of year effect (^∗^ if *p*-value < 0.05; ^∗∗^ if *p*-value < 0.01, and ^∗∗∗^ if *p*-value < 0.001).*

*The significance of the year effect is indicated for the three variables that were observed in a second experiment (Experiment 2). See text for abbreviation meaning.*

The architectural traits (*Nb_Leave*s, *Trunk_length*, and *Nb_syll*) and MAppleT parameters (*IN_length*, *Leaf_area*, *RLE*_GDD_**, and *loga_syll_*) showed intermediate (0.38) to high (0.86) heritabilities in both experiments (**Table 1**). The highest heritability values were found for *IN_length* in Experiment 1. Heritabilities for *Trunk_length* were similar whether computed considering the two experiments together or for each year separately. Conversely, heritability values were reduced by 26 and 32% for *Nb_Leave*s and *Nb_syll*, when considering either both years together or each year separately.

The analyses of correlations gave similar results if genetic or phenotypic correlations were considered (**Table 2**). Correlations between MAppleT parameters were low. Only the correlations between RLE*_GDD_* and the mean internode length and between the leaf area and the mean internode length were significant (*P* = 0.004 and 0.0003, respectively). As expected, *Nb_leaves*, *Trunk_length*, and *Nb_syll* were strongly correlated to *RLE*_GDD_**, *IN_length*, and *loga_syll_*, respectively. The correlations between the two experiments for the architectural traits *Nb_Leaves*, *Trunk_length*, and *Nb_syll* were significant but the correlation coefficients remained low (from 0.25 to 0.45).

**Table 2:** Correlation between phenotypic (lower diagonal) and genetic values (upper diagonal) of either measured traits (*Nb_Leaves*, *Trunk_length*, *Nb_Syll*, *IN_length*, and *Leaf_area*) or estimated parameters (*RLE*_GDD_**, *loga_syll_*) on 116 1-year-old hybrid apple trees in the two experiments (Experiments 1 and 2).

	Experiment 1							Experiment 2		
		
	Nb_Leaves	Trunk_length	Nb_Syll	IN_length	Leaf_area	RLE*_GDD_*	log*a_syll_*	Nb_Leaves	Trunk_length	Nb_Syll
**Experiment 1**										
Nb_Leaves	1	**0.41**	**0.25**	-**0.21**	-0.06	**0.77**	0.08	**0.25**		
Trunk_length	**0.55**	1	-0.03	**0.80**	**0.27**	0.16	-0.03		**0.45**	
Nb_Syll	**0.30**	0.09	1	-**0.19**	0.16	**0.28**	**0.84**			**0.32**
IN_length	-0.09	**0.77**	-0.11	1	**0.32**	-**0.32**	-0.08			
Leaf_area	0.08	**0.26**	0.10	**0.24**	1	-0.03	0.07			
RLE*_GDD_*	**0.60**	**0.21**	**0.28**	-**0.18**	-0.003	1	0.07			
log*a_syll_*	**0.15**	0.06	**0.81**	-0.04	0.05	0.05	1			
**Experiment 2**										
Nb_Leaves	**0.25**							1	**0.68**	**0.42**
Trunk_length		**0.45**						**0.72**	1	0.08
Nb_Syll			**0.32**					**0.41**	**0.15**	1


*Significant correlations according to Pearson’s test are indicated in bold.*

### Genomic Prediction and Its Accuracy

QTL detections on BLUPs of *IN_length*, *Leaf_area*, *RLE*_GDD_**, and *loga_syll_* revealed only one QTL for *IN_length* which was the most heritable parameters (data not shown). This QTL was located on LG 12 and explained 16.9% of the genetic variability. It was associated with the SNP FB_0152374_L12_PA with a LOD score of 4.67. For the three other variables (*RLE*_GDD_**, *loga_syll_*, and *Leaf_area*), no significant QTL could be detected. Because these variables were under genetic control (moderate to high heritability), the absence of detected QTL led us to assume that they were under the control of a high number of markers with low effect. We therefore tested a genome wide prediction model.

**FIGURE 2:**
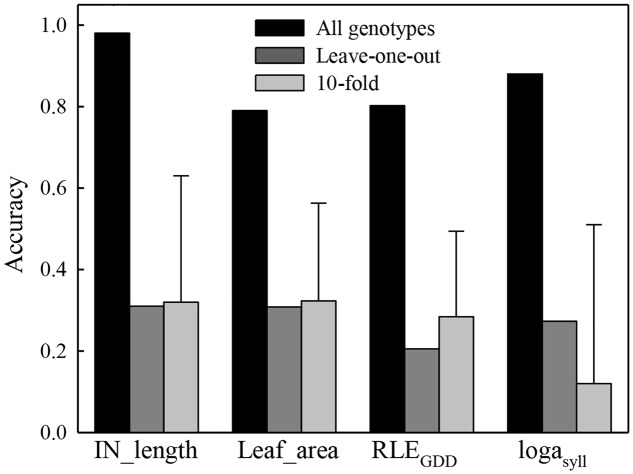
**Accuracy of the genome wide predictions obtained using 116 1-year-old hybrid apple trees of a segregating population as training set, with a Leave-one-out cross validation and a 10-fold cross validation for four parameters (*IN_length*, *Leaf_area*, *RLE*_GDD_**, and *loga_syll_*) of the MAppleT model.** The error bar for the 10-fold cross validation was computed from the 10 values of correlations between the observed and predicted value of the validation population. For each fold, 90% of the population was randomly chosen as the training population and the other 10% were used as the validation population. For the leave-one-out cross validation, each phenotype was predicted using marker effects estimated on the whole population excluding this genotype. The accuracy was calculated once as the correlation between predicted and observed phenotypes for the whole population.

Correlations between mean observed phenotypes and predicted phenotypes with the genome wide estimation model and using all genotypes to estimate marker effects was 0.82 for *RLE*_GDD_**, 0.98 for *IN_length*, 0.87 for *loga_syll_*, and 0.80 for *Leaf_area*, respectively (**Figure 2**). All these correlations were significant according to Pearson’s test (*P* < 0.05) showing the ability of the genome wide model to estimate parameter values from SNP markers. However, the prediction accuracies were notably lower using the cross validation methods (**Figure 2**). The mean accuracy from the 10-fold cross validation was 0.26 for *RLE*_GDD_**, 0.30 for *IN_length*, 0.15 for *loga_syll_*, and 0.31 for *Leaf_area*, respectively. The standard deviation of the accuracy from the 10-fold cross validation was very high for the four variables: 0.23 for *RLE*_GDD_**, 0.30 for *IN_length*, 0.35 for *loga_syll_*, and 0.24 for *Leaf_area*. The accuracy obtained using the “leave-one-out” cross validation were 0.20 for *RLE*_GDD_**, 0.30 for *IN_length*, 0.25 for *loga_syll_*, and 0.30 for *Leaf_area*.

### Simulation of 1 Year-Old Genotypes under Two Environmental Conditions

**FIGURE 3:**
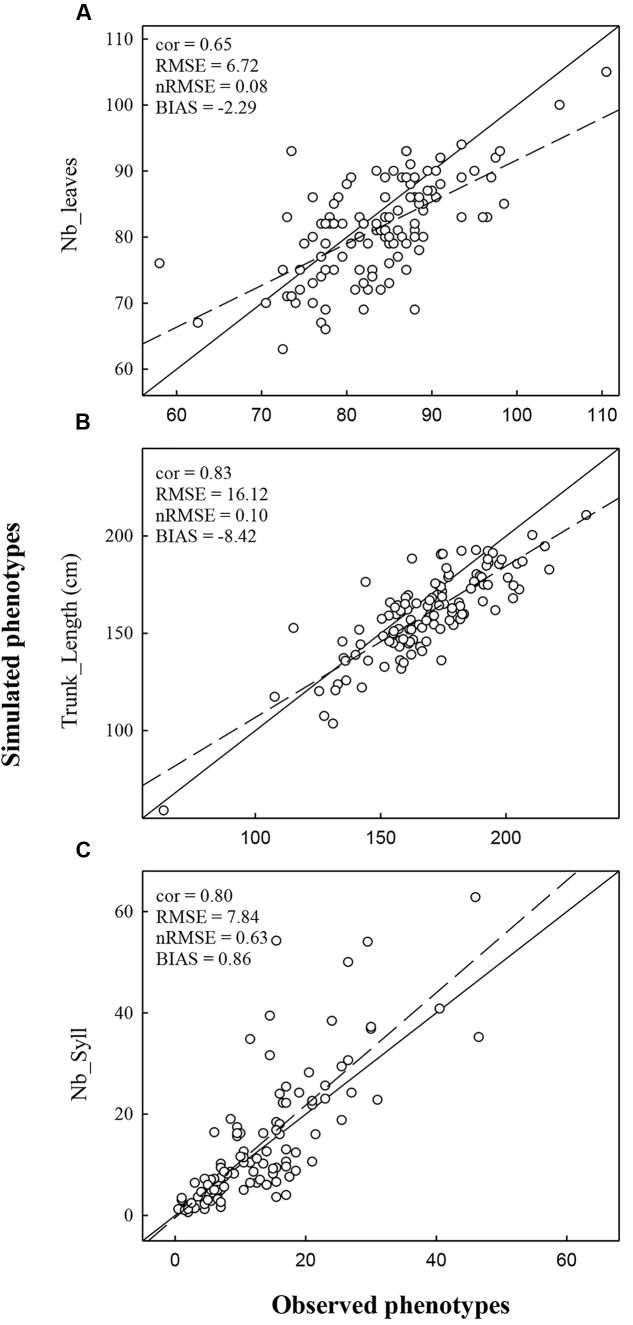
**Comparison of observed and simulated phenotypes for the variable *Nb_Leaves*** **(A)**, *Trunk_length* **(B)**, and *Nb_Syll* **(C)**. Simulations were performed using climatic conditions of Experiment 1. The dashed line represents the regression line and the full line represents the 1:1 line.

**FIGURE 4:**
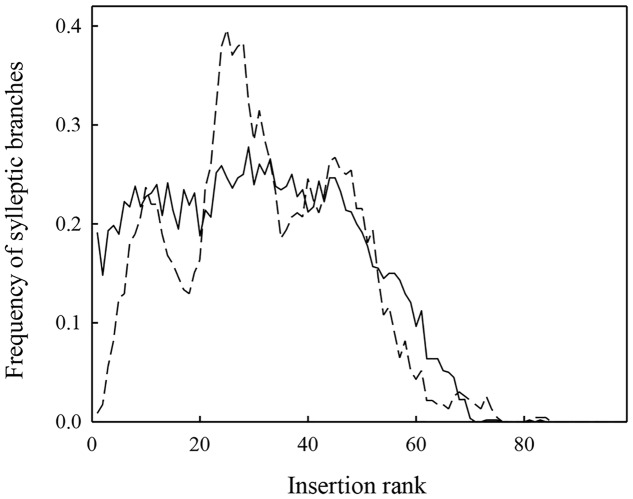
**Distribution of the mean frequency of sylleptic laterals along the trunks for 116 1-year-old apple trees either simulated (full line) or observed (dashed line) in Experiment 1**.

Simulations with MAppleT were performed under environmental conditions of Experiments 1 and 2. Under the environmental conditions of Experiment 1 (experiment used to parameterize MAppleT), correlations between observed and simulated values were equal to 0.65 for *Nb_Leaves*, 0.83 for *Trunk_Length*, and 0.80 for *Nb_syll* (**Figure 3**). These correlations were significant according to Pearson’s test (*P* < 0.05). The normalized RMSE (*nRMSE*) was low for the *Nb_leaves* (8%) and *Trunk_length* (10%) but high for *Nb_syll* (63%). The *Nb_syll* was correctly predicted for the genotypes with low number of sylleptic laterals but the difference between observation and simulation increased for genotypes with more than 10 sylleptic laterals. On average, the number of sylleptic was slightly overestimated (by 0.86). Conversely, *Nb_leaves* and *Trunk_length* were on average underestimated by 2.29 and 8.42 mm, respectively. The distribution of sylleptic laterals along the trunk was more homogeneous in the simulated population than in the observed one (**Figure 4**). Indeed, a large variation in this frequency that was not simulated by the model was observed along trunk between 10 and 30 nodes. However, the highest frequency of sylleptic laterals was obtained within the same internode rank zone (∼ at rank 25) and the decrease after node rank 40 was correctly simulated.

**FIGURE 5:**
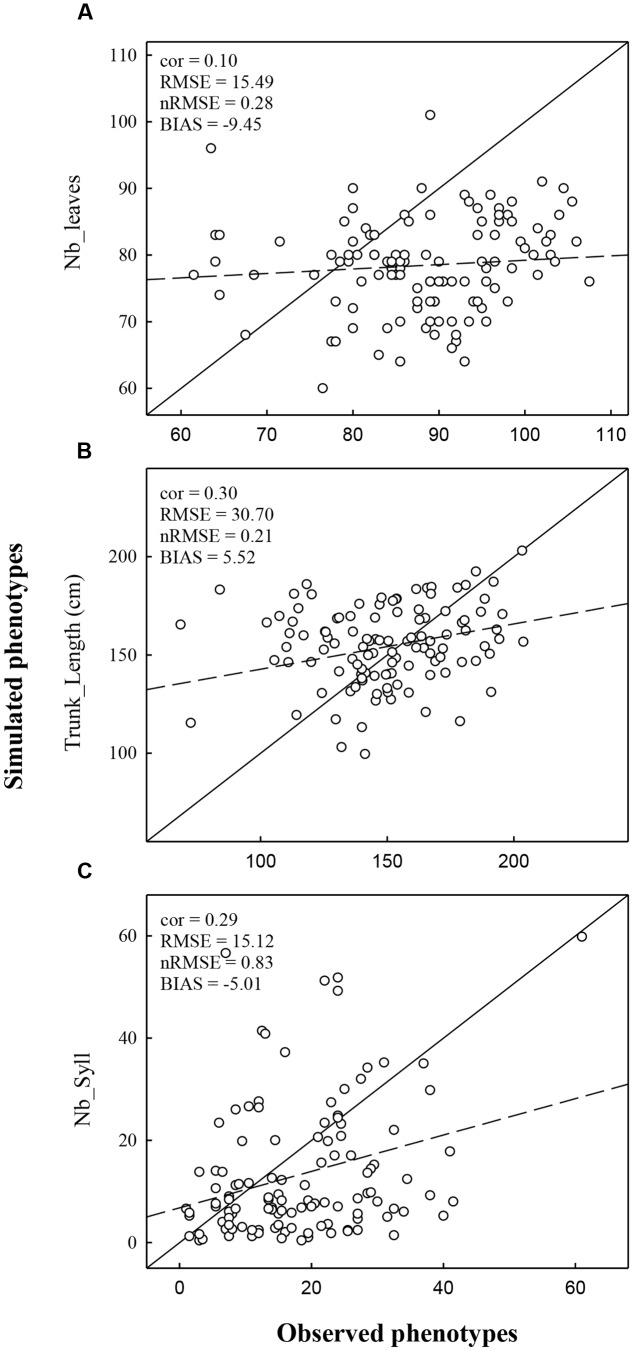
**Comparison of observed and simulated phenotype for the variable *Nb_Leaves*** **(A)**, *Trunk_length* **(B)**, and *Nb_Syll* **(C)**. Simulations were realized using climatic conditions of Experiment 2. The dashed line represents the regression line and the full line represents the 1:1 line.

The performance of the model was also evaluated by comparing observed and predicted values obtained from simulations under the environmental condition of Experiment 2 which was not used to estimate marker effects. In Experiment 2, correlations between observed and simulated values were lower than in Experiment 1 (**Figure 5**). Correlations were low but significant for *Trunk_length* (0.30) and *Nb_syll* (0.29). The correlation was not significant for *Nb_Leaves* (0.10). The *nRMSE* obtained for *Nb_leaves*, *Trunk_length*, and *Nb_syll* were 20, 21, and 83%, respectively.

The total number of leaves was more strongly underestimated in Experiment 2 than Experiment 1 (bias was -9.45 and -2.29, respectively). This discrepancy resulted from the average number of leaves observed in Experiments 1 and 2 (83.57 and 87.86, respectively) which were not in accordance with the lower cumulate thermal time observed in Experiment 2 than in Experiment 1. Despite of the strong underestimation of the number of internodes (corresponding to the number of leaves), the trunk length was overestimated in Experiment 2, unlike Experiment 1. This probably results from an overestimation of the mean individual internode length in Experiment 2. Indeed, mean internode length in MAppleT was identical in the two simulations whereas their observed values were higher in Experiment 1 than in Experiment 2 (2.02 and 1.69 cm, respectively). The number of sylleptic laterals was highly underestimated in Experiment 2 (bias was -5.01). This is probably due to the underestimation of the leaf emergence rate which in turn affects the probability of sylleptic emergence (Equation 1).

**FIGURE 6:**
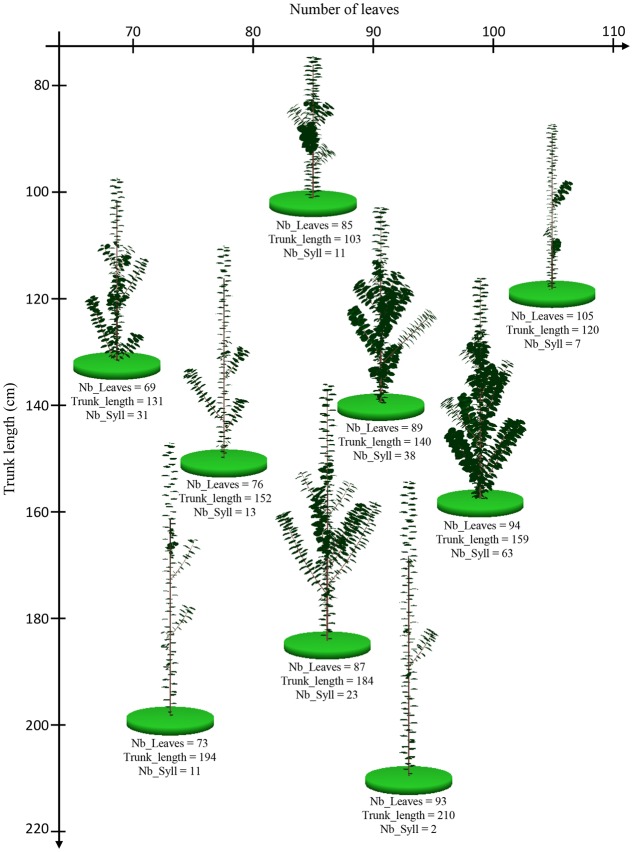
**Graphical outputs of MAppleT simulations for nine apple tree genotypes with contrasting architectures.** The position of trunk base of the virtual trees on each axis of the graphic indicates their number of leaves on the trunk (Nb_Leaves, *X*-axis) and the trunk length (Trunk_length, *Y*-axis), respectively. The values of the number of leaves, trunk length, and number of sylleptic laterals (Nb_Syll) are indicated under each virtual tree.

The 1-year-old apple trees simulated in the environmental condition of Experiment 1 exhibited a large range of phenotypes. Genotypes with high number of leaves and high trunk length or with low number of leaves and high trunk length were simulated (**Figure 6**). This suggests low correlations between the simulated traits. Indeed, the simulated architectural traits were slightly correlated (**Table 3**) with correlation coefficients similar to those observed (**Table 2**). The correlations between the architectural traits obtained from simulations in the two climatic conditions were close to 1 (**Table 3**) probably showing that the model did not generate large GxE interaction.

**Table 3:** Correlations between architectural traits simulated with MAppleT and genotypic marker effects for 116 1-year-old hybrid apple genotypes in two experimental climatic conditions.

	Experiment 1			Experiment 2		
		
	Nb_Leaves	Trunk_length	Nb_Syll	Nb_Leaves	Trunk_length	Nb_Syll
**Experiment 1**						
Nb_Leaves	1	**0.34**	**0.26**	**0.99**		
Trunk_length		1	-0.09		**0.99**	
Nb_Syll			1			**0.98**
**Experiment 2**						
Nb_Leaves				1	**0.35**	**0.26**
Trunk_length					1	-0.10
Nb_Syll						1


*Significant correlations according to Pearson’s test are indicated in bold.*

## Discussion

This paper presents a proposition for linking a FSPM, MAppleT, with genetic information on growth and branching model parameters, estimated on an apple tree segregating population. Mean heritability values of architectural traits (trunk length, number of leaves, and number of sylleptic laterals) were estimated with two independent datasets which provided similar results. However, heritability values were lower than those obtained by Segura et al. (2006), on a subsample of 50 hybrids of the same progeny, observed at the same age (1-year-old) but planted in a different year (2003) and with three replicates per genotype (Segura et al., 2006). Even though heritability values were similar in Experiments 1 and 2, both phenotypic and genetic correlations between these experiments were low. This suggests that the proportion of phenotypic variation explained by the genotype is constant but also that the genetic values vary between years. This could result from differences in the climatic conditions in the plantation years, from the variability in the plant material obtained after grafting and from the existence of large GxE interactions. This GxE interaction has been previously observed in clonally propagated plants (Alspach and Oraguzie, 2002; Chagné et al., 2014). Nevertheless, having re-multiplied the same progeny several times is quite unique for a perennial crop in which a single orchard and a low number of replicates per genotype are often considered. Our results suggest that part of the GxE interaction could result from propagation effect and confirm that more attention should be paid in the future to the phenotypes repeatability, through multi-sites or multi-years experiments, in order to improve our understanding of the potential sources of variability (Myles et al., 2009).

Previous studies aiming at linking FSPM with genetic information have been based on QTL effects only (Xu et al., 2011). Here, the marker effects were estimated first with interval mapping methods but a few QTLs could be detected. This contrasts with previous studies (Segura et al., 2007, 2009) and could result from a lack of precision of either genotypes or phenotypes and from the large GxE interaction.

To overcome this, our strategy was to perform a genome wide estimation of marker effects for the four studied parameters. Correlations between estimated and observed values from the genome wide prediction model were high, showing that the model was able to estimate genotypic differences from SNP polymorphisms. However, during the cross validation, the accuracy was much lower even though similar to previous studies on apple (Kumar et al., 2012; Muranty et al., 2015) or on other species (Rutkoski et al., 2013; Fodor et al., 2014). This highlights the limit of our genome wide prediction model to predict phenotypic values for genotypes that are not included in the training set. The prediction model accuracy is influenced by the density of the genetic map (Kumar et al., 2012; Heslot et al., 2013), the population size (Brito et al., 2011), and the heritability of traits (Combs and Bernardo, 2013; Muranty et al., 2015). Among the arguments mentioned above, the population size is probably the most limiting factor in the present study. In that context, high throughout methods such as airborne imagery (Virlet et al., 2014) or 3D laser scanning (Boudon et al., 2014) could be useful to provide phenotypic data related to plant functioning or architecture on large sets of genotypes. Other statistical models used to predict markers effects could also be tested. Bayesian models that include a selection of variables together with a shrinkage parameter for marker effects could be tested in order to reduce the risk of over-fitting. Indeed, this over-fitting problem could explain the low ability of our modeling approach to predict genotypes that were not included in the training set (de los Campos et al., 2013). Nevertheless, even if these methods could be relevant, several studies have compared the predictions for the different statistical methods and have shown similar accuracy whatever the method used (e.g., Resende et al., 2012; Fodor et al., 2014).

The predicted values of four MAppleT parameters (*IN_length*, *Leaf_area, RLE*_GDD_**, and *loga_syll_*) were used to simulate tree development with MAppleT and three integrative traits (*Nb_Leaves*, *Trunk_length*, and *Nb_syll*) were considered for validating our approach. The RMSE was low for the three variables when the genotypes were simulated in climatic conditions of Experiment 1, used to parameterize the genome wide model. This demonstrates the feasibility and relevance of the approach, i.e., including genetic information into a FSPM. As expected, the number of sylleptic laterals was correctly simulated for Experiment 1, consistently with the fact that this variable was used to estimate the probability of sylleptic lateral emergence. Moreover, the node ranks along the trunks at which the frequency of sylleptic laterals was the highest was similar to observations. The decrease in sylleptic frequency after node rank 40 was also consistent with observations. This supports the assumption we made of a close relationship between the RLE and the sylleptic branching probability. However, the sylleptic laterals were more homogeneously distributed along the trunks in the simulated population than in observations. This suggests that the link between the RLE and the sylleptic branching could be stronger or more complex than modeled in this study. Further refinements in the link between the dynamics of primary growth and immediate branching, considering non-linear relationships between RLE and branching probability (Génard et al., 1994), could be considered.

Consistently with the discrepancy between the number of leaves and the GDD in the two observation years, the comparison of the integrated variables simulated and observed with the climatic conditions of Experiment 2 generated high RMSE. As previously stated for peach tree by Davidson et al. (2015), our results confirm that modeling the leaf emergence rate based on GDD is not sufficient, in particular for trees grown in field conditions. Other environmental factors are likely to affect the RLE in open field conditions, such as the soil resource availability. Since the emergence of sylleptic laterals depended in our model on the leaf emergence rate only, discrepancy was also obtained for this variable when simulated with Experiment 2 conditions.

Despite these limitations and the necessity of further improvements of the predictions based on genome wide information, the MAppleT model including the genotypic marker effects simulate genotypes with contrasting architectures for which correlations among simulated architectural traits were similar to observations. This suggests that the different hybrid genomes corresponding to different trait recombination were correctly taken into account. The existence of numerous recombination of architectural traits in F1 hybrids and the difficulties that this generates for the qualitative assessment of hybrid architecture was previously underlined by Segura et al. (2009). The presented approach thus opens a new way to experiment the consequences of genome recombination on integrated phenotypes. However, further evaluation on the agronomic performance of each genotype when the tree will be mature would be useful to complement the present study. Even though quantitative genetic models allows studying the genetic determinism of several traits jointly though multi-traits approaches (e.g., Mathews et al., 2008; Alimi et al., 2013), the exploration of several, possibly not correlated, traits remains a challenge to facilitate the exploration of plant ideotypes (Martre et al., 2015). Indeed, ideotypes are by definition composed of several desirable traits. FSPMs, by providing simulations of plant architecture and allowing the investigation of the impact of architectural traits on integrative traits such as light interception or biomass production (e.g., Da Silva et al., 2014) constitute a powerful tool to assess phenotype performance.

The integration of other environmental factors in the present modeling approach would be necessary to extent the domain of validity and to further analyze GxE interactions. This could be achieved by integrating other environmental response curves and adding the effects of each individual environmental factor on plant growth processes as done for instance by Reymond et al. (2003), Hammer et al. (2006), or Casadebaig et al. (2011). Alternative approaches including an intermediate plant variable that takes into account the combined effects of many environmental factors could be relevant. In this way, functional structural plant models considering the impact of C source-sink balances on plant and organ growth through a variable (the index of competition for carbon) reflecting the internal trophic state of the plant have been proposed (e.g., Luquet et al., 2006; Mathieu et al., 2009; Pallas et al., 2013). Such approaches could be appropriate to reduce the risk of overestimating the environmental effects that result from the additive models used in above mentioned studies (Reymond et al., 2003; Hammer et al., 2006; Casadebaig et al., 2011). In this present study, the RLE could be considered as an intermediate variable since it determines the branching probability. Presently, RLE was affected by temperature, only but its use as an intermediate variable could be tested under other environmental constraints such as water stress.

Finally, the extension of such models coupling FSPM and genetic information to larger germplasms and to the generation of a new simulated mapping population, as recently proposed in rice (Xu and Buck-Sorlin, 2016), will likely be a new research avenue in interface with eco-physiology, process-based, and gene-based modeling (Technow et al., 2015). For perennials, the extension to multi-years development of trees will represent another complexity that could be tackled based on knowledge on genetic determinisms over years (Segura et al., 2008, 2009).

## Author Contributions

EC and BP contributed to design the work. VM and BP performed the modeling tasks and analyzed the data. VM, BP, and EC wrote the manuscript. EC contributed to data acquisition.

## Conflict of Interest Statement

The authors declare that the research was conducted in the absence of any commercial or financial relationships that could be construed as a potential conflict of interest.
