# Supplementary material for: Combining Genome-Wide Information with a Functional Structural Plant Model to Simulate 1-Year-Old Apple Tree Architecture
Source: Front Plant Sci. 2017 Jan 12;7:2065. doi: 10.3389/fpls.2016.02065 (PMC5226960; doi:10.3389/fpls.2016.02065)
Supplement: Supplementary file 1 [file Image_1.pdf]

Supplementary Material

Combining genome-wide information with functional structural plant model to simulate 1-year-old apple tree architecture

Vincent Migault[1], Benoît Pallas[1] and Evelyne Costes[1,*]

[1]INRA, UMR AGAP, Equipe Architecture et Fonctionnement des Espèces Fruitières, F-34398 Montpellier, France

* **Correspondence**: costes@supagro.inr.fr

1. Supplementary material

1.1. Analysis of temperature effect on the rate of leaf emergence

The effect of temperature on the rate of leaf emergence (RLE) and the genetic effect on the response of RLE to temperature were analysed. In order to evaluate the significance of these effects, nine linear models (presented in Suplementary Table 1) were fitted on data from Exp 1 (11 dates of measurement from early July until end of September) and the models were compared according to the Bayesian information criterion (BIC). Assuming a normal distribution of RLE, the models can take into account a linear influence of the thermal time on RLE (*a* and *b* for an effect on the intercept or slope, respectively) and a genetic effect on this linear response (α_i *and* β_i for the effect on the intercept or slope, respectively). No thermal time effect was included in the first two models which assumed constant RLE during growing season but model 2 includes a genetic effect on RLE unlike model 1. The seven other models take into account a linear relation between RLE and thermal time. The intercept of the linear response was assumed to be zero in models 3 and 4. In model 4, the slope was assumed to be genetically dependent unlike model 3. In model 5, the linear response was considered as identical for all genotypes. In model 6, the intercept was considered as identical for all genotypes but the slope was assumed to be genetically dependent. Conversely, the slope was considered as identical for all genotypes but the intercept was assumed to be genetically dependent in model 7. In models 8 and 9, both the intercept and the slope were assumed to be genetically dependent.

The response of RLE to thermal time was modelled in two different ways, considering either the daily mean temperature (T_{Mean}) or the single sine method (T_{SSM}) using 7 and 35°C as lower and upper thresholds, respectively, as proposed in Lescourret et al. (1998) for peach tree. The models were fitted on experimental data with the *lmer* procedure of the *lme4* package of R software.

The models which do not take into account the temperature effect (models 1 and 2) present higher BIC than the other ones (see Supplementary Table 2). This demonstrates the significance of temperature effect on RLE. The best model according to BIC was model 4 which used the single sine method for computing thermal time (TT). Consequently, with this model 4, the intercept of the linear relation between RLE and TT was equal to 0 and the slope was considered to be genetically dependent. This slope, noted RLE_{GDD}, corresponds to the rate of leaf emergence expressed in growing degree-days.

Supplementary Table 1. Equations of the different linear models used to model the temperature and genotypic effects on the rate of leaf emergence.

Model	Equation	Degrees of freedom
Model 1	$RLE_{ij} = a + \epsilon_{ij}$ with $\epsilon_{ij} \sim \mathcal{N}(0, \sigma_\beta)$	2
Model 2	$RLE_{ij} = (a + \alpha_i) + \epsilon_{ij}$ with $\alpha_i \sim \mathcal{N}(0, \sigma_\beta)$, $\epsilon_{ij} \sim \mathcal{N}(0, \sigma_\beta)$	3
Model 3	$RLE_{ij} = b * X_j + \epsilon_{ij}$ with $\epsilon_{ij} \sim \mathcal{N}(0, \sigma_\beta)$	2
Model 4	$RLE_{ij} = (b + \beta_i) * X_j + \epsilon_{ij}$ with $\beta_i \sim \mathcal{N}(0, \sigma_\alpha)$, $\epsilon_{ij} \sim \mathcal{N}(0, \sigma_\beta)$	3
Model 5	$RLE_{ij} = a + b * X_j + \epsilon_{ij}$ with $\epsilon_{ij} \sim \mathcal{N}(0, \sigma_\beta)$	3
Model 6	$RLE_{ij} = (a + \alpha_i) + b * X_j + \epsilon_{ij}$ with $\alpha_i \sim \mathcal{N}(0, \sigma_\alpha)$, $\epsilon_{ij} \sim \mathcal{N}(0, \sigma_\beta)$	4
Model 7	$RLE_{ij} = a + (b + \beta_i) * X_j + \epsilon_{ij}$ with $\beta_i \sim \mathcal{N}(0, \sigma_\beta)$, $\epsilon_{ij} \sim \mathcal{N}(0, \sigma_\beta)$	4
Model 8	$RLE_{ij} = (a + \alpha_i) + (b + \beta_i) * X_j + \epsilon_{ij}$ with $\alpha_i \sim \mathcal{N}(0, \sigma_\alpha)$, $\beta_i \sim \mathcal{N}(0, \sigma_\beta)$, $\epsilon_{ij} \sim \mathcal{N}(0, \sigma_\beta)$	5
Model 9	$RLE_{ij} = (a + \alpha_i) + (b + \beta_i) * X_j + \epsilon_{ij}$ with $\begin{pmatrix} \alpha_i \\ \beta_i \end{pmatrix} \sim \mathcal{N}\left(\begin{pmatrix} 0 \\ 0 \end{pmatrix}, \begin{pmatrix} \sigma_\alpha & \sigma_{\alpha\beta} \\ \sigma_{\alpha\beta} & \sigma_\beta \end{pmatrix} \right)$, $\epsilon_{ij} \sim \mathcal{N}(0, \sigma_\beta)$	6

In equations, RLE_{ij} is the rate of leaf emergence observed for the ith genotype during the jth week, X_j is the thermal time observed during the jth week, a and b are the mean intercept and slope of the population, respectively, and α_i and β_i are the genetic effects on intercept and slope, respectively, for the ith genotype, ε_{ij} are the Gaussian residuals of the model.

Supplementary Table 2. Values of BIC obtained for the different linear models considering either the daily mean temperature (T_{Mean}) or the thermal time computed with the single sine method (TT).

criteria	Model 1	Model 2	Model 3		Model 4		Model 5	
			T_{Mean}	T_{SSM}	T_{Mean}	T_{SSM}	T_{Mean}	T_{SSM}
BIC	-2218	-2221	-2480	-2486	-2485	**-2493**	-2490	-2478

criteria	Model 6		Model 7		Model 8		Model 9	
	T_{Mean}	T_{SSM}	T_{Mean}	T_{SSM}	T_{Mean}	T_{SSM}	T_{Mean}	TT
BIC	-2490	-2478	-2491	-2480	-2483	-2472	-2475	-2464

Bold value indicates the best model according to the BIC

1.2. Sylleptic branching model and estimation of model parameters

1.2.1 Description of the model

At day d, the probability of sylleptic lateral appearance (P_d) on the trunk was assumed to be linearly dependent to the rate of leaf emergence (*RLE*) observed during the n_{days} previous days. Moreover, we assumed that lateral sylleptic budburst can happen at a given rank (called r_k) below the terminal apex, only. In the model this probability was applied when a metamer was produced by the terminal meristem, only. If we consider k as the rank of the last emitted metamer, the probability of producing a sylleptic lateral at rank k-r_k can be formalized by the following equation:

$$P_d(sylleptic\ branching\ (k - rk)|Phytomer\ creation\ (k)) = a_{syll} * \frac{1}{n_{days}} \sum_{i=d-n_{days}}^{d} RLE_i$$

where a_{syll} is the coefficient of the linear relation between the probability of sylleptic lateral appareance and the mean value of RLE_d during the previous n_{days} days (the rate of leaf emergence at day d).

From this equation, the probability of sylleptic lateral appearance at day d can be expressed as follows:

$$P_d(sylleptic\ branching)$$

$$= P_d(sylleptic\ branching\ (k - LD)|Phytomer\ emergence\ (k)) * P_d(Phytomer\ emergence\ (k))$$

$$= a_{syll} * \left(\frac{1}{n_{days}} \sum_{i=d-n_{days}}^{d} RLE_i\right) * RLE_d$$

1.2.2 Estimation of a_{syll}

a_{syll} was estimated from the total number of sylleptic (N_{syll}) produced on each tree during experiment 1. If we considered d_b as the day at which the first sylleptic lateral on the tree can appear and d_e the day of the end of the growing season, the sum of P_d between both days is an approximation of N_{syll}.

$$N_{syll} \approx \sum_{d=d_b}^{d_e} P_d(sylleptic\ branching)$$

d_b was considered as the date when the phytomer of rank r_k was produced by the tree. The date of appearance of this phytomer was estimated from the value of RLE_{GDD} and from the thermal time from budburst.

The previous equations can be expressed as

$$N_{syll} = \sum_{d=d_b}^{d_e} a_{syll} * \left(\frac{1}{n_{days}} \sum_{i=d-n_{days}}^{d} RLE_i \right) * RLE_d$$

$$N_{syll} = a_{syll} * \sum_{d=d_b}^{d_e} (RLE_{GDD})^2 * \left(\frac{1}{n_{days}} \sum_{i=d-n_{days}}^{d} TT_i \right) * TT_d$$

Finally, the value of a_{syll} can be estimated as follows:

$$a_{syll} = \frac{N_{syll}}{(RLE_{GDD})^2 * \sum_{d=d_b}^{d_e} \left(\frac{1}{n_{days}} \sum_{i=d-n_{days}}^{d} TT_i \right) * TT_d}$$

In this equation TT_i is computed from meteorological data, d_e and RLE_{GDD} are model parameters and N_{syll} is obtained from experimental data. Since the values of all of these parameters are known, the value of a_{syll} can be estimated for each tree of the experiment.

2. Supplementary figures

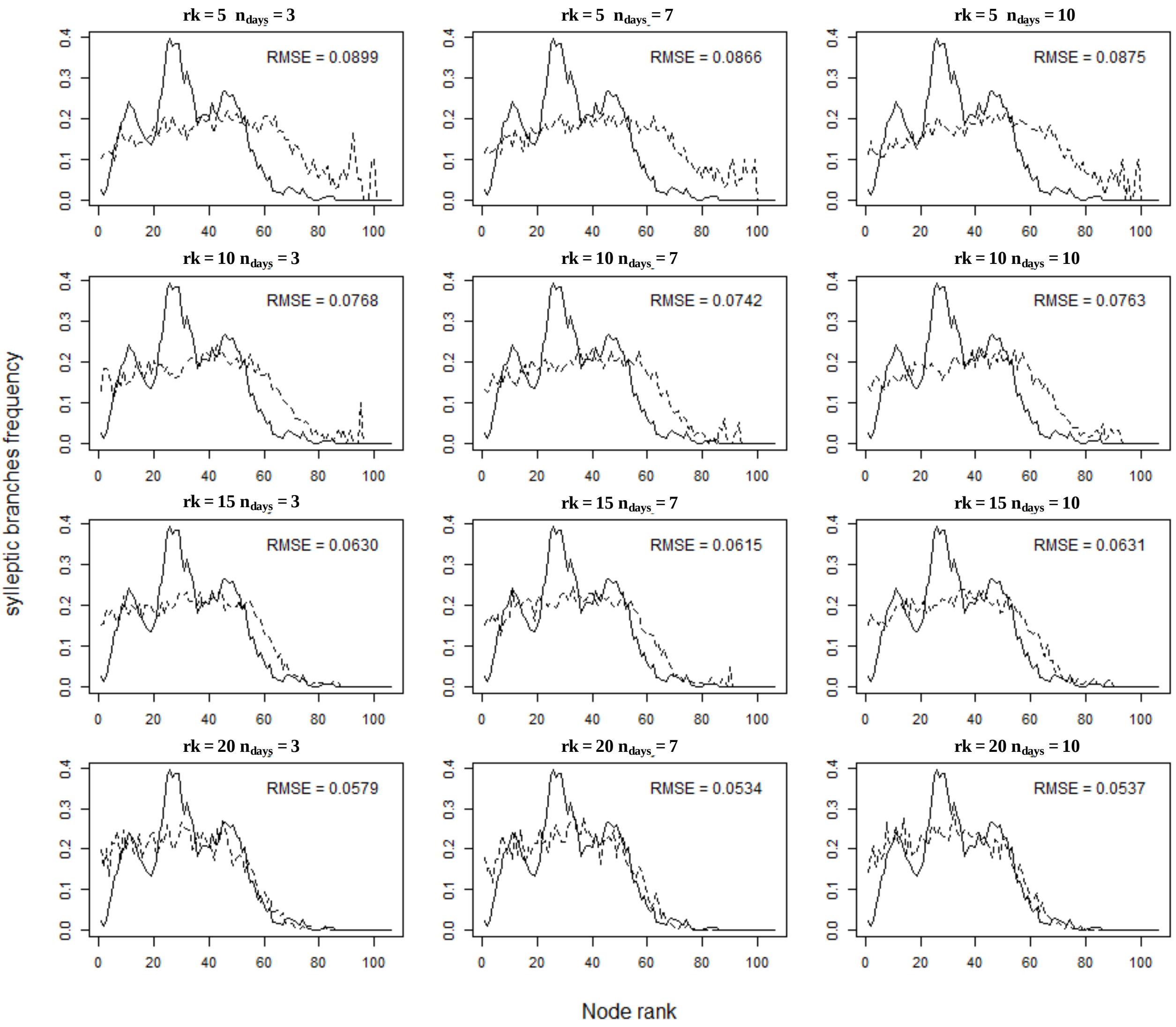

Supplementary Figure 1. Observed (full line) and simulated (dashed line) distribution of the mean frequency of sylleptic laterals along trunks with different values of r_k (5, 10, 15 and 20) and n_{days} (3, 7 and 10). The root mean squared errors (RMSE) between observed and simulated distributions are indicated for each combination of parameter values.
